# Effect of MAFLD on albuminuria and the interaction between MAFLD and diabetes on albuminuria

**DOI:** 10.1111/1753-0407.13501

**Published:** 2023-11-16

**Authors:** Yufang Liu, Sanbao Chai, Xiaomei Zhang

**Affiliations:** ^1^ Department of Endocrinology Peking University International Hospital Beijing China

**Keywords:** abnormal albuminuria, diabetes, hepatic fibrosis, MAFLD, reduced eGFR

## Abstract

**Objective:**

To investigate the effects of metabolic associated fatty liver disease (MAFLD) on chronic kidney disease (CKD) and abnormal albuminuria and the interaction between MAFLD and diabetes on abnormal albuminuria.

**Methods:**

Data of participants in the American 2017–2018 National Health and Nutrition Examination Survey were analyzed. Hepatic steatosis was defined as median controlled attenuation parameter ≥248 dB/m, which was measured by ultrasound transient elastography. MAFLD was defined by evidence of hepatic steatosis on ultrasound in addition to any metabolic dysregulation. Hepatic fibrosis was detected by FibroScan and quantified by parameter of stiffness (E). Hepatic fibrosis was defined as E ≥ 9.7 kPa. As component of CKD, reduced estimated glomerular filtration rate (eGFR) was defined as<60 mL/min/1.73 m^2^ and abnormal albuminuria was defined as urinary albumin‐to‐creatinine ratio ≥ 30 mg/g.

**Results:**

Data pertaining to 5119 participants were included in the analysis, with 40.6% hepatic normal, 52.1% MAFLD, and 7.2% hepatic fibrosis. Multivariable regression analyses showed that for abnormal albuminuria, the odds ratio (OR) was 0.82 (0.65–1.04) for MAFLD group and 1.73 (1.14.–,2.63) for hepatic fibrosis group, both taking the hepatic healthy group as reference. As for reduced eGFR, the OR was 0.68 (0.51–0.92) for MAFLD group and 0.93 (0.56–1.53) for hepatic fibrosis group. Diabetes was significantly related to greater risk of abnormal albuminuria (3.04 [2.70–3.42]) and reduced eGFR (1.53 [1.33–1.77]). With regard to the prevalence of abnormal albuminuria, the OR was 1.64 (1.03–2.60) for those with hepatic fibrosis only, 3.30 (2.80–3.89) for those with diabetes only, and 5.05 (3.30–7.72) for those with both two conditions. But there were neither additive interaction (relative excess risk due to interaction 0.56 [−1.41–.53], *p* = .577) nor multiplicative interaction (OR 0.81 [0.45–1.47], *p* = .492) between hepatic fibrosis and diabetes on the prevalence of abnormal albuminuria.

**Conclusion:**

MAFLD with hepatic fibrosis is an independent risk factor for abnormal albuminuria, but it does not have interaction with diabetes on abnormal albuminuria.

## Graphical abstract

**Highlights**

- Data of 5119 participants from the American 2017–2018 National Health and Nutrition Examination Survey were analyzed.
- In our study, hepatic steatosis was measured by ultrasound transient elastography and hepatic fibrosis was detected by FibroScan.
- It was revealed that metabolic associated fatty liver disease (MAFLD) with hepatic fibrosis is an independent risk factor for abnormal albuminuria, whereas MAFLD without hepatic fibrosis is not associated with abnormal albuminuria.
- MAFLD with or without hepatic fibrosis was not associated with CKD.
- There were neither additive interaction nor multiplicative interaction between hepatic fibrosis and diabetes on the prevalence of abnormal albuminuria.

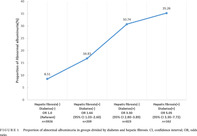

## 1 INTRODUCTION

Nonalcoholic fatty liver disease (NAFLD) and chronic kidney disease (CKD) are two chronic diseases with increasing prevalence, affecting nearly 30% and 15% of the general adult population, respectively.^1^, ^2^ There have been studies reported that NAFLD is a risk factor for the development of incident CKD.^3^, ^4^ Regardless of the coexisting metabolic diseases, such as obesity, hypertension, type 2 diabetes mellitus (T2DM) or metabolic syndrome, high severity of NAFLD can increase the risk of CKD.^5^, ^6^ Meta‐analysis found that NAFLD (detected by multiple methods) is associated with up to 40% increase in the long‐term risk of incident CKD.^6^ To define fatty liver disease more precisely, MAFLD was raised by an international panel of experts from 22 countries in early 2020.^7^ Recently, the definition of MAFLD was confirmed to be more practical and accurate compared to NAFLD at identifying patients with hepatic steatosis at high risk of liver disease progression, such as liver fibrosis and liver cancer.^8^

Recently, it was reported that MAFLD and MAFLD with increased liver fibrosis score are closely and independently related to CKD and abnormal albuminuria.^9^ Although there have been a number of studies explored the association between MAFLD and CKD, the results were inconclusive. Meanwhile, as a proved independent risk factor for abnormal albuminuria, diabetes is also a very common comorbidity of MAFLD. However, few studies explored the interaction between diabetes and MAFLD on abnormal albuminuria to date. Therefore, the aim of this study is to investigate the effects of MAFLD on reduced estimated glomerular filtration rate (eGFR) and abnormal albuminuria and the interaction between MAFLD and diabetes on abnormal albuminuria.

## 2 METHODS

### 2.1 Population

The National Health and Nutrition Examination Survey (NHANES) is a population‐based, cross‐sectional survey designed to gather information about the health and nutrition of the US household population. Each year, the project surveys a nationally representative sample of about 5000 people across the country. The NHANES interview section includes questions related to demographics, socioeconomics, diet, and health. The physical examination part includes physiological measurements, laboratory tests, and so on. The survey was approved by the National Center for Health Statistics institutional review board and all subjects signed written informed consent.

Data of subjects in the American NHANES 2017–2018 survey cycle were analyzed. NHANES data are publicly available and can be accessed online (https://www.cdc.gov). Participants with missing relevant data and lack of relevant examinations were excluded from the analyses. The analyses of present study were limited to adult individuals, which means all participants were 18 years of age or older.

Race in the present study was categorized into white, black, Mexican, Asian, and other races and ethnicities. Body mass index (BMI) was calculated by dividing body weight (kg) by square of height (m). NHANES provides data for three consecutive blood pressure (BP) measurements and we used the second one to avoid deviation caused by emotional tension, physical activities or other factors that can make influence on blood pressure.

### 2.2 Definition of reduced eGFR and abnormal albuminuria

We calculated eGFR using the Chronic Kidney Disease Epidemiology Collaboration equation,^10^ which is as follows: eGFR = 141 × min (Scr/κ, 1)^α^ × max (Scr/κ, 1)^−1.209^ × 0.993^Age^ × 1.018 (if female), where Scr is serum creatinine, κ is 0.7 for females and 0.9 for males, α is −0.329 for females and − 0.411 for males, min indicates the minimum of Scr/κ or 1, and max indicates the maximum of Scr/κ or 1. Urinary albumin‐to‐creatinine ratio (ACR) was calculated by dividing the urinary albumin (mg) by urinary creatinine (g). In the present study, reduced eGFR was defined as eGFR<60 mL/min/1.73 m^2^ and abnormal albuminuria was defined as ACR≥30 mg/g.^11^

### 2.3 Definition of diabetes

The oral glucose tolerance test is to measure the plasma glucose value 2 hours after oral administration of 75 g glucose. In the present study, subjects accord with any of the following conditions were diagnosed as diabetic patients: (a) confirmed history of diabetes in questionnaire; (b) glycosylated hemoglobin (HbA1c) level ≥6.5%; and (c) fasting glucose level ≥7.0 mmol/L.^12^ Participants eligible for all of the following conditions were classified as normal glucose tolerance: (a) denied history of diabetes or prediabetes in questionnaire; (b) HbA1c level <5.7%; and (c) fasting glucose level <5.6 mmol/L.

### 2.4 Definition of MAFLD and hepatic fibrosis

Hepatic steatosis was quantified by the parameter of controlled attenuation parameter (CAP), which was measured by ultrasound transient elastography.^13^ The ultrasound attenuation reflects the presence of hepatic steatosis and is recorded as the CAP. As a result, CAP is an indicator for fat accumulation in the liver. In this study, hepatic steatosis was defined as CAP ≥248 dB/m.^14^, ^15^ MAFLD was defined by evidence of hepatic steatosis detected by ultrasound in addition to any of the following conditions: overweight/obesity, T2DM, or metabolic dysregulation.^7^, ^9^ Hepatic fibrosis was detected by FibroScan, which applied ultrasound and the vibration controlled transient elastography (VCTE) to derive liver stiffness.^16^ Hepatic fibrosis was quantified by parameter of stiffness (E), and in the present study, hepatic fibrosis was defined as E ≥ 9.7 kPa.^17^

### 2.5 Statistical analysis

The NHANES used a complex, multistage, probability sampling design to select participants representative of the civilian, non‐institutionalized US population, so we take this into account in our analyses by using sample weights to adjust for the unequal probability of selection into the survey and to adjust for the possible bias resulting from nonresponse according to NHANES analytic guidelines. The Kolmogorov–Smirnov method was used to evaluate the data distribution. Continuous variables were represented as mean ± SD for normally distributed data or medians (interquartile ranges) for abnormally distributed data. Chi‐square test, Mann–Whitney *U* test, or independent *t* test was applied to compare the differences between two groups when appropriate. Categorical variables were represented as frequency (percentage) and chi‐square test was performed to evaluate the between‐group differences. Logistic regression was performed to adjust for potential confounders when appropriate. *P* value <.05 was considered indicative of statistical significance. All statistical analyses were performed using STATA 17.0.

## 3 RESULTS

Baseline characteristics of the study population are summarized in Table 1. Data pertaining to 5119 participants were included in the analysis. Among them, 2079 subjects (40.6%) were hepatic normal (without MAFLD or hepatic fibrosis). Three hundred seventy‐one subjects (7.2%) had both MAFLD and hepatic fibrosis. The remaining 2669 individuals, who had MAFLD but no hepatic fibrosis, accounted for 52.1%. As expected, compared to people without MAFLD and those with MAFLD but without hepatic fibrosis, individuals in the hepatic fibrosis group were significantly older and had higher BMI, alanine aminotransferase, aspartate aminotransferase, total triglyceride, serum creatinine, ACR, and uric acid (UA) but lower level of high‐density lipoprotein cholesterol. In the hepatic fibrosis group, the proportion of males was higher (60.4%), so was diabetes (43.7%). There was no significant difference in systolic blood pressure (SBP) levels among the three groups (*p* = .168).

**TABLE 1:** Characteristics of the Study Population by MAFLD and hepatic fibrosis.

	MAFLD (−) hepatic fibrosis (−) (*n* = 2079)	MAFLD (+) hepatic fibrosis (−) (*n* = 2669)	MAFLD (+) hepatic fibrosis (+) (*n* = 371)	*p* value
Sex (male, %)	919 (44.2)	1391 (52.1)	224 (60.4)	<.001
Age (years)	44.9 ± 19.5	52.3 ± 17.0	56.1 ± 15.8	<.001
Race or ethnicity (%)				
White	700 (33.7)	897 (33.6)	146 (39.4)	<.001
Black	560 (26.9)	536 (20.1)	81 (21.8)	
Hispanic	390 (18.8)	713 (26.7)	91 (24.5)	
Asian	314 (15.1)	388 (14.5)	31 (8.4)	
Other	115 (5.5)	135 (5.1)	22 (5.9)	
BMI (kg/m^2^)	25.6 ± 5.3	31.6 ± 6.5	37.5 ± 10.3	<.001
SBP (mmHg)	121.7 ± 20.2	128.6 ± 19.5	134.3 ± 19.1	.168
Diabetes (%)	166 (8.0)	657 (24.6)	162 (43.7)	<.001
ALT (U/L)	18.4 ± 15.6	24.0 ± 15.4	34.0 ± 31.4	<.001
AST (U/L)	20.5 ± 10.3	21.9 ± 10.8	32.1 ± 30.1	<.001
Scr (umol/L)	78.2 ± 32.7	79.1 ± 40.2	90.3 ± 71.0	<.001
ACR (mg/g)	6.92 (4.52–12.56)	7.76 (5.00–15.32)	10.39 (5.81–29.89)	<.001
TG (mmol/L)	1.23 ± 0.83	1.89 ± 1.49	1.93 ± 1.41	<.001
TC (mmol/L)	4.72 ± 1.01	4.95 ± 1.06	4.73 ± 1.13	.008
HDL‐c (mmol/L)	1.49 ± 0.39	1.29 ± 0.37	1.25 ± 0.43	.001
UA (μmol/L)	300.1 ± 80.9	338.5 ± 85.9	366.6 ± 101.1	<.001

Abbreviations: ACR, albumin creatinine ratio; ALT, alanine aminotransferase; AST, aspartate aminotransferase; BMI, body mass index; HDL‐c, high‐density lipoprotein cholesterol; MAFLD, metabolic associated fatty liver disease; SBP, systolic blood pressure; Scr, serum creatinine; TC, total cholesterol; TG, triglyceride; UA, uric acid.

To further understand the association between MAFLD and kidney damage (reduced eGFR and abnormal albuminuria), we performed univariable and multivariable logistic regression analyses stratified by MAFLD and hepatic fibrosis, as shown in Table 2. As for the prevalence of abnormal albuminuria, after adjusting for age, sex, race, diabetes, BMI, SBP, and UA, the OR was 0.82 (0.65–1.04) for MAFLD group and 1.73 (1.14–2.63) for hepatic fibrosis group, both taking hepatic healthy group as reference. The OR was 1.42 (1.03–1.95) for hepatic fibrosis group, when taking MAFLD as reference group. In conclusion, hepatic fibrosis was associated with greater risk of abnormal albuminuria, whereas MAFLD was not related to the prevalence of abnormal albuminuria. With regard to the prevalence of reduced eGFR, after adjusting for confounding factors, the OR was 0.68 (0.51–0.92) for MAFLD group and 0.93 (0.56–1.53) for hepatic fibrosis group, both taking hepatic healthy group as reference. The OR was 1.36 (0.91–2.02) for hepatic fibrosis group, when taking MAFLD as reference group. In conclusion, MAFLD was associated with lower risk of reduced eGFR, whereas hepatic fibrosis had no association with reduced eGFR.

**TABLE 2:** Effect of MAFLD and hepatic fibrosis on abnormal albuminuria and reduced eGFR.

		MAFLD (+) hepatic fibrosis (−)		MAFLD (−) hepatic fibrosis (−)	MAFLD (+) hepatic fibrosis (+)		MAFLD (+) hepatic fibrosis (−)	MAFLD (+) hepatic fibrosis (+)	
	MAFLD (−) hepatic fibrosis (−)	OR (95% CI)	*p* value		OR (95% CI)	*p*		OR (95% CI)	*p* value
Abnormal albuminuria									
Model 1	Ref.	1.35 (1.13–1.61)	.001	Ref.	2.80 (2.12–3.70)	<.001	Ref.	2.08 (1.60–2.71)	<.001
Model 2	Ref.	1.11 (0.92–1.34)	.257	Ref.	2.15 (1.60–2.88)	<.001	Ref.	1.95 (1.49–2.56)	<.001
Model 3	Ref.	0.82 (0.65–1.04)	.108	Ref.	1.73 (1.14–2.63)	.010	Ref.	1.42 (1.03–1.95)	.032
Reduced eGFR									
Model 1	Ref.	1.16 (0.95–1.42)	.156	Ref.	2.19 (1.60–3.02)	<.001	Ref.	1.89 (1.39–2.57)	<.001
Model 2	Ref.	1.00 (0.79–1.26)	.992	Ref.	1.70 (1.18–2.45)	.005	Ref.	1.68 (1.19–2.37)	.003
Model 3	Ref.	0.68 (0.51–0.92)	.011	Ref.	0.93 (0.56–1.53)	.768	Ref.	1.36 (0.91–2.02)	.134

*Note*: Model 1 was unadjusted (univariate); Model 2 was adjusted for age, sex, and race; Model 3 was adjusted for model 2 adjustments plus diabetes, body mass index, systolic blood pressure, and uric acid.

Abbreviations: CI, confidence interval; eGFR, estimated glomerular filtration rate; MAFLD, metabolic associated fatty liver disease; OR, odds ratio; Ref., reference.

As we expected, no matter in univariate model or multivariable adjusted model, diabetes was significantly related to greater risk of abnormal albuminuria (3.04 [2.70–3.42]) and reduced eGFR (1.53 [1.33–1.77]), as we can see in Table 3. In conclusion, diabetes was an independent risk factor for both abnormal albuminuria and reduced eGFR.

**TABLE 3:** Effect of diabetes on abnormal albuminuria and reduced eGFR.

	Abnormal albuminuria			Reduced eGFR		
	NGT	Diabetes		NGT	Diabetes	
		OR (95% CI)	*p* value		OR (95% CI)	*p* value
Model 1	Ref.	4.26 (3.95–4.59)	<.001	Ref.	3.87 (3.53–4.24)	<.001
Model 2	Ref.	2.93 (2.68–3.21)	<.001	Ref.	1.92 (1.73–2.14)	<.001
Model 3	Ref.	3.04 (2.70–3.42)	<.001	Ref.	1.53 (1.33–1.77)	<.001

*Note*: Model 1 was unadjusted (univariate). Model 2 was adjusted for age, sex, and race. Model 3 was adjusted for model 2 adjustments plus body mass index, systolic blood pressure, and uric acid.

Abbreviations: CI, confidence interval; eGFR, estimated glomerular filtration rate; NGT, normal glucose tolerance; OR, odds ratio; Ref., reference.

**FIGURE 1:**
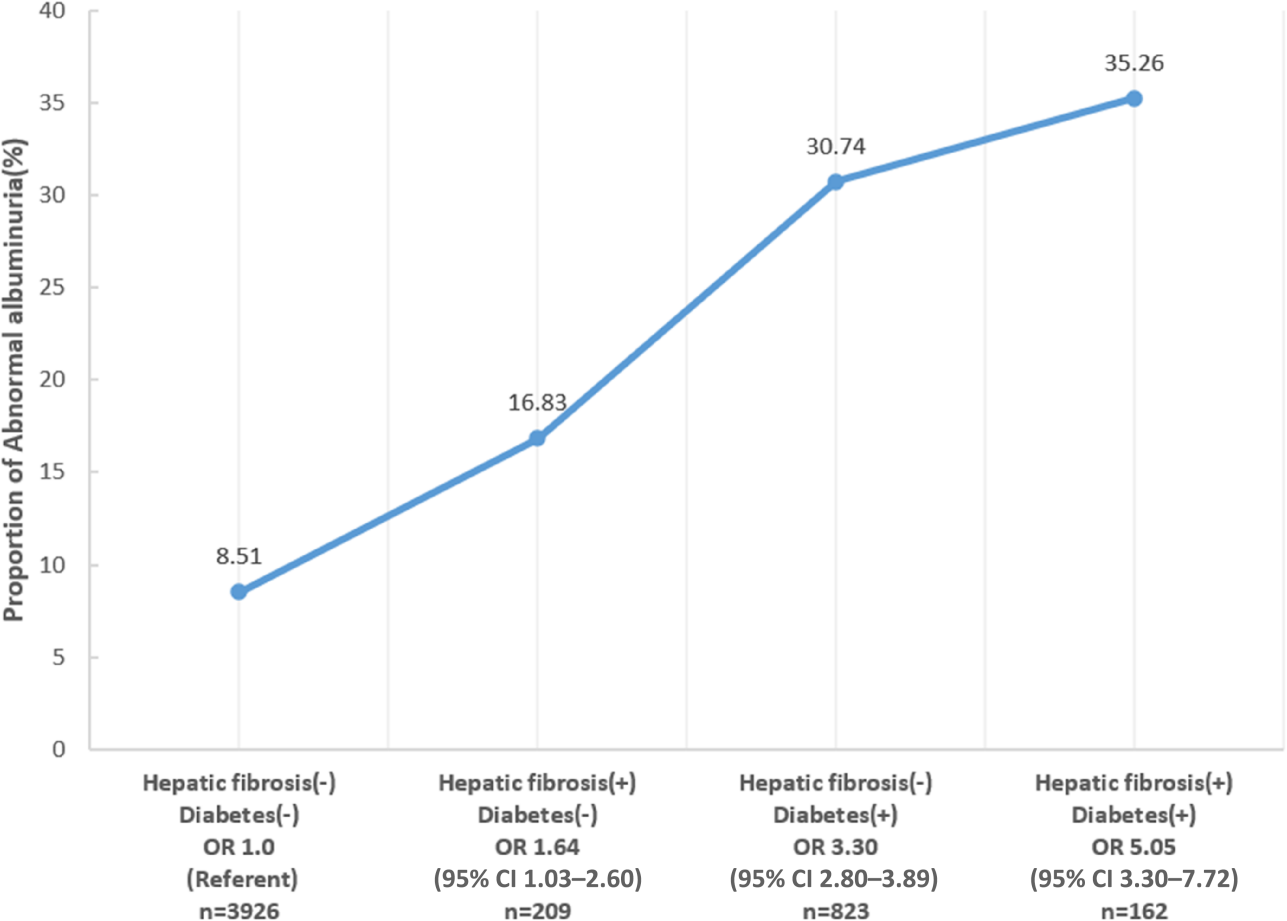
Proportion of abnormal albuminuria in groups divided by diabetes and hepatic fibrosis. CI, confidence interval; OR, odds ratio.

For the prevalence of abnormal albuminuria, when taking those without diabetes or hepatic fibrosis as reference, the OR was 1.64 (1.03 –2.60) for those with hepatic fibrosis only, 3.30 (2.80–3.89) for those with diabetes only, and 5.05 (3.30–7.72) for those with both two conditions (Figure 1 and Table 4). As both hepatic fibrosis and diabetes were significantly associated with greater risk of abnormal albuminuria, we further performed interaction analysis between hepatic fibrosis and diabetes. There were neither additive interaction (relative excess risk due to interaction 0.56 [−1.41 to 2.53], *p* = .577) nor multiplicative interaction (OR 0.81 [0.45–1.47], *p* = .492) between hepatic fibrosis and diabetes on the prevalence of abnormal albuminuria (Table 4).

**TABLE 4:** Interaction (additive and multiplicative) between diabetes and hepatic fibrosis on abnormal albuminuria.

Abnormal albuminuria	Hepatic fibrosis (−)	Hepatic fibrosis (+)	Additive interaction	Multiplicative interaction
	OR (95% CI) *p* value	OR (95%CI) *p* value	RERI (95% CI) *p* value	OR (95% CI) *p* value
Diabetes (−)	1 (Ref.)	1.64 (1.03 to 2.60) *p* = .036	0.56 (−1.41 to 2.53) *p* = 0.577	0.81 (0.45 to 1.47) *p* = .492
Diabetes (+)	3.30 (2.80 to 3.89) *p*<.001	5.05 (3.30 to 7.72) *p*<.001		

*Note*: Adjusted for age, sex, race, body mass index, systolic blood pressure, and uric acid.

Abbreviations: CI, confidence interval; OR, odds ratio; Ref., reference; RERI, relative excess risk due to interaction.

## 4 DISCUSSION

Previous meta‐analysis of 19 studies showed the risk of albuminuria among patients with NAFLD was significantly higher than those without NAFLD,^18^ whereas our study indicated that MAFLD without fibrosis was not related to the prevalence of abnormal albuminuria. Different conclusion was derived due to the different definition of NAFLD and MAFLD. On the other hand, in our study, we divided MAFLD subjects into two groups according to the presence or absence of liver fibrosis and analyzed them separately, which was different from previous studies.

With the same study population, a recent study by Stefano Ciardullo et al revealed that liver fibrosis but not liver steatosis was associated with albuminuria, and neither liver steatosis nor liver fibrosis was associated with reduced eGFR.^19^ On the basis of this study, we further restricted research object to patients with MAFLD instead of simply liver steatosis patients, in consideration of the closely connection between MAFLD and diabetes. A recent meta‐analysis containing seven cross‐sectional studies revealed that liver stiffness measured by VCTE is significantly associated with CKD and albuminuria in patients with NAFLD.^20^ Besides the replacement of NAFLD to MAFLD, the different founding could also due to the distinct definition of CKD and reduced eGFR. As CKD represent a wider spectrum of disease than reduced eGFR, association observed in patients with reduced eGFR may not exist in CKD population, which was confirmed by previous study.^19^ Our study revealed that hepatic fibrosis is associated with abnormal albuminuria but not with reduced eGFR, probably due to that albuminuria is well known to be the early clinical manifestation of diabetic nephropathy whereas decline in eGFR occurs in late stage of diabetic kidney disease. In addition to that, eGFR would even temporarily increase in very early stage of diabetic nephropathy. The difference between groups would diminish or disappear when eGFR has declined.

Previous study showed that MAFLD and MAFLD with increased liver fibrosis score are strongly and independently associated with CKD as well as abnormal albuminuria.^9^ This differs from the conclusions of our study, partly due to the different populations studied. The aforesaid study used the NHANES III database, with a timespan from 1988 to 1994, whereas our study used NHANES 2017–2018 database. The difference also partly due to the different definition of hepatic fibrosis. Aforesaid study employed noninvasively estimation by using NAFLD fibrosis score and fibrosis 4 score to assess liver fibrosis, whereas the present study used liver stiffness derived by FibroScan to quantify hepatic fibrosis. By using transient elastography, our present study has the advantage of quantitative measurements validated in different populations, as well as the high reproducibility to detect liver fibrosis. These two differences may lead to differences in conclusions, and further validation will require larger studies or meta‐analyses.

A study from Asia reported liver fibrosis was independently associated with early kidney disease, defined as the presence of microalbuminuria with an eGFR≥60 mL/min/1.73 m^2^.^4^ Another Asian study reported that advanced hepatic fibrosis but not steatosis was independently associated with an increased risk of albuminuria≥30 mg/g.^21^ Our study further validates this conclusion. Individuals with hepatic fibrosis have a greater risk of abnormal albuminuria, whether compared with individuals without MAFLD or individuals with MAFLD but no fibrosis.

About the mechanisms that linked MAFLD, T2DM, and CKD, there were several hypotheses: (a) clustering of metabolic risk factors coexisting with metabolic syndrome, insulin resistance, dyslipidemia, and hypertension^22^, ^23^, ^24^; (b) platelet activation and the release of multiple proinflammatory cytokines, chemokines, and growth factors that accelerate progression of MAFLD and CKD^5^; and (c) intestinal dysbiosis, resulting in increased gram‐negative bacterium, lipopolysaccharides, secondary bile acids, intestinal permeability, and renal toxins.^25^, ^26^

In fact, many of the risk factors for MAFLD with fibrosis have the potential to influence the development of abnormal albuminuria. First, most scholars believe that MAFLD with liver fibrosis may aggravate systemic and liver insulin resistance, and insulin resistance can release a variety of mediators that promote inflammation, coagulation, oxidation, and fibrosis, thus worsening kidney hemodynamics and leading to kidney disease.^27^, ^28^, ^29^, ^30^ Second, the renin‐angiotensin system may contribute to liver and kidney disease progression by increasing ectopic lipid deposition, proinflammatory cytokine production, and promoting insulin resistance. Furthermore, against the background of increased risk for cardiovascular disease commonly seen in patients with MAFLD, the potential for endothelial dysfunction and renal vascular damage may also become more prominent.^31^ Last but not least, increased oxidative stress upregulates the transcription of various antioxidant and detoxification enzymes through nuclear factor erythroid‐2 related factor 2, leading to liver fat deposition and renal impairment.

As we expected, no matter in univariate model or multivariable adjusted model, diabetes was significantly related to greater risk of abnormal albuminuria and reduced eGFR. The coexistence of MAFLD and diabetes is very common, and both hepatic fibrosis and diabetes are considered independent risk factors for abnormal albuminuria, so it is important to explore the interaction between these two conditions.

Interaction comprises additive interaction and multiplicative interaction. In this study, two interactions were analyzed for the effects of hepatic fibrosis and diabetes on abnormal albuminuria.

Subgroup analysis of a meta‐analysis demonstrated the significantly increased risk of albuminuria among patients with NAFLD but no diabetes, whereas no significant association between albuminuria and NAFLD among diabetic patients.^18^ The results of this subgroup analysis suggest that the effect of NAFLD on albuminuria is influenced by diabetes, that is, NAFLD and diabetes may have an interaction in their effect on albuminuria. In this study, we found that patients with hepatic fibrosis with coexisting diabetes had a higher prevalence of abnormal albuminuria than their counterparts without diabetes or without hepatic fibrosis. However, we examined the interaction between liver fibrosis and diabetes on abnormal albuminuria from two perspectives, and the results showed that there was neither additive interaction nor multiplicative interaction. Perhaps due to the limitation of sample size, we did not find any interaction between these two conditions, leading this part of the conclusion to be verified by subsequent larger sample studies.

It is an advantage that our study has a large sample size and a unique prospective cohort of MAFLD patients with detailed documentation of clinical and biochemical information. We were able to systematically adjust for potential confounders including age, sex, race, BMI, SBP, and UA, which were known factors potentially affecting the onset and progression of abnormal albuminuria.^32^, ^33^, ^34^ Diabetes is a confirmed risk factor for abnormal albuminuria, and diabetes and MAFLD often coexist. There have been many studies on the relationship between MAFLD and abnormal albuminuria or early kidney disease; however, few studies have paid attention to the interaction between MAFLD and diabetes in the onset and progression of abnormal albuminuria. Although our study has obtained negative results in this regard, it can provide new ideas and inspiration for future research.

## AUTHOR CONTRIBUTIONS

Yufang Liu and Sanbao Chai conceived and designed the experiments. Yufang Liu performed the data analysis. Yufang Liu wrote the manuscript. Xiaomei Zhang provided supervision. All authors contributed to the article and approved the submitted version.

## FUNDING INFORMATION

These investigators received no specific grant from any funding agency in the public, commercial or not‐for‐profit sectors.

## CONFLICT OF INTEREST STATEMENT

The authors declare that the research was conducted in the absence of any commercial or financial relationships that could be construed as a potential conflict of interest.

## ETHICS STATEMENT

This study protocol of NHANES was reviewed and approved by the National Center for Health Statistics Research Ethics Review Board. The approval numbers for the protocols were Protocol #2011‐17 and Protocol #2018‐01. Signed informed consent was obtained from all participants.
